# Intranasal Ion-Triggered In Situ Delivery System of Virus-like Particles: Development Using the Quality by Design Approach

**DOI:** 10.3390/polym16050685

**Published:** 2024-03-02

**Authors:** Elena O. Bakhrushina, Iosif B. Mikhel, Valeriya M. Kondratieva, Irina M. Zubareva, Svetlana I. Kosenkova, Anastasiya V. Belyatskaya, Olga I. Stepanova, Ivan I. Krasnyuk, Tatyana V. Grebennikova, Ivan I. Krasnyuk

**Affiliations:** 1 A.P. Nelyubin Institute of Pharmacy, I.M. Sechenov First Moscow State Medical University (Sechenov University), Moscow 119048, Russia; bakhrushina_e_o@staff.sechenov.ru (E.O.B.); kashlikova_i_m@staff.sechenov.ru (I.M.Z.); kosenkova_s_i@staff.sechenov.ru (S.I.K.); belyatskaya_a_v@staff.sechenov.ru (A.V.B.); stepanova_o_i@staff.sechenov.ru (O.I.S.); krasnyuk_i_i_1@staff.sechenov.ru (I.I.K.J.); krasnyuk_i_i@staff.sechenov.ru (I.I.K.); 2 Departament of Epidemiology and Microbiology, The Gamaleya National Centre of Epidemiology and Microbiology, Moscow 123098, Russia; 1999valeriak@mail.ru (V.M.K.); t_grebennikova@mail.ru (T.V.G.)

**Keywords:** in situ forming, VLP, intranasal delivery, vaccines, quality by design, ion-triggered polymers, gellan gum

## Abstract

The rapid growth in the prevalence of infectious diseases requires timely action from drug developers. In recent years, the COVID-19 pandemic has demonstrated the unpreparedness of the population for such emergencies. The introduction of modern methods of Design of Experiments (DoE) is required to accelerate the process of drug development and bring a drug to market. The main objective of this study was to develop an ion-triggered in situ system for intranasal delivery of VLP using a Quality by Design approach. Based on a literature review and initial studies, the key QTPP, CQA, CPP, and CMA were identified to develop a novel delivery system for virus-like particles. As a result of the studies on the quality attributes of the developed delivery system, an ion-triggered in situ gel meeting all the specified parameters was obtained using the Quality by Design method.

## 1. Introduction

In situ systems (ISS) are a new generation of drug delivery systems that undergo a phase transition (sol–gel transition) at the site of application. The sol–gel transition is influenced by various physiological and pathological conditions within the body, including temperature, ionic composition, pH, humidity, and the diffusion of solvents into the surrounding soft tissues. In addition to stimuli within the organism that cause in situ phase transitions, it is possible to apply external influences—UV and IR radiation—to form in situ gels and implants. So-called smart polymers are known to exhibit such phase-transitioning properties.

Smart polymers are classified according to the stimulus for in situ gel formation. The most famous representatives are thermoreversible polymers, which form gels when the temperature rises. On the other hand, there are pH-sensitive polymers that undergo a sol–gel transition when the pH changes significantly. The ion-triggered polymers undergo a phase transition when interacting with ions in the surrounding mucosa and tissues, forming an “egg box” structure. In the case of phase-inversion, the stimulus for gel formation is the solvent changing—when the solvent from the system diffuses into the surrounding tissues, an insoluble matrix is formed at the injection site. Photosensitive smart polymers are able to form stable gels and implants under IR and UV irradiation. Moisture-activated ones swell and form gels when interacting with water molecules (the most common are powders). Depending on the desired localization of the drug application site, it is necessary to select a group of smart polymers with a specific sol–gel transition stimulus.

Ion-triggered polymers in ISS technology find application in the development of delivery systems for use on mucosa with a wide range of ionic compositions of biological fluids. Therefore, one of the most commonly realized routes of administration of ion-triggered polymer-based systems is intranasal administration [1,2,3].

Nasal fluid has a pH of approximately 6.0 and an approximate ionic composition that can be reproduced in vitro using a solution of NaCl, KCl, and CaCl_2_ (15:5:1) [4].

The ion-triggered polymers used in ISS technology are united by their origin and the gel structures they form. These polymers are often natural components derived from vegetable and microbial products. They are non-starch polysaccharides such as alginates, carrageenans, gums, pectin, etc. [5]. All non-starch polysaccharides are capable of absorbing large amounts of water and usually form gels in the presence of bivalent ions.

Another factor that unites ion-triggered polymers is the presence of their own pharmacological properties. This property is used by researchers to enhance the main therapeutic effect of the delivery system [6].

Thus, pectins and alginates have reliable hypocholesterolemic and hypotriglyceridemic effects. Experimental studies and clinical observations have demonstrated the therapeutic efficacy of pectins in infectious pathology, manifested in the reduction of intoxication [7]. 

The main problem with gelling agents, which are non-starch polysaccharides, is a significant difference in their properties depending on the growing conditions and characteristics of the initial raw materials, methods of their processing, purification, and technology of polymer extraction. The initial polysaccharides may have different molecular weights, viscosities, water solubilities, and degrees of esterification and sulphation, which directly affect not only the technological parameters of the system, but also the biological activity of carbohydrate biopolymers. The lack of generally accepted quantitative indicators for standardization of non-starch polysaccharide preparations makes it difficult to carry out a comparative assessment of the pharmacological efficacy of polysaccharides among themselves, as well as with drugs of similar action [8,9]. 

The in situ gelation stimulus of gellan gum (GG)-based formulations is identical to that of alginates and pectins. Compared to other ion-selective polymers, GG does not require a high concentration of divalent ions at the mucosal surface to initiate the sol–gel transition. Several studies have shown experimentally that in situ GG-based gels are stable enough to withstand the harsh conditions of the nasal cavity. Gellan gum is a natural polysaccharide produced by the bacterium Sphingomonas elodea. The main problem with natural polymers is the difficulty in standardizing and purifying them. In a review of studies on the development of delivery systems based on GG and other natural polymers, gum has always shown good results, regardless of the manufacturer. Other natural polymers, on the other hand, varied in quality performance when the manufacturer, raw material base, or method of obtaining the polymer was changed [6,10,11,12,13].

To sum up, the main advantages of ion-triggered polymers include instant gelation requiring a moderate amount of gelling agent, biocompatibility and biodegradability, good mucoadhesion, low pregelation viscosity, and a pronounced phase transition. At the same time, many authors have noted [14,15] the high sensitivity of ion-triggered polymers during final sterilization, indicating the need for researchers to closely study the stability of polymer matrices before and after sterilization.

Although the advantages of ion-triggered in situ polymers allow them to be used as monocomponent matrices, it is possible to add mucoadhesive polymers to increase the binding strength of the matrix to the mucosa.

The most common example of a mucoadhesive polymer is chitosan [16]. Although chitosan has some mucoadhesive properties in its native state, its biocompatibility and release capacity are limited due to its rapid degradation in the body. Because of these properties of chitosan, its configurations and salts are widely used [17,18]. In addition to chitosan, various cellulose modifications such as methyl cellulose (MC), hydroxyethyl cellulose (HEC), hydroxypropyl methyl cellulose (HPMC), and hydroxypropyl cellulose (HPC) are commonly used [19,20]. A less well-known mucoadhesive polymer is poloxamer 124 (Pol 124). A study by Yong, C. S. et al. demonstrated the mucoadhesive properties of poloxamers 124 and 188 in the development of new drugs [21,22].

As mentioned above, ion-triggered ISSs are particularly common in the development of drugs for intranasal administration. The use of intranasal in situ systems avoids most of the problems associated with the administration of standard nasal dosage forms: loss of drug dose, rapid removal of the dosage form from the surface of the nasal mucosa, and enzymatic degradation of the drug by mucosal enzymes. In the pharmaceutical development of intranasal in situ systems, thermoreversible, pH-sensitive, and ion-selective in situ polymers are most frequently used [10,12,13]. In addition, it should be noted that phase-sensitive matrices, which are commonly used in in situ system technologies for other routes of administration, are obviously not suitable for intranasal delivery of immunobiological drugs (IBDs) because they involve an organic solvent that diffuses into soft tissues, which may interfere with natural mucociliary clearance, the protective functions of the nasal cavity, as well as the patient’s sense of smell. Despite the high gelation rate of moisture-activated polymers, their use for intranasal administration may be inappropriate due to the protective mechanisms of the nasal cavity—irritation of the mucosa by the powder composition results in sneezing and rapid removal of the drug from the site of application.

The high compatibility of ion-triggered polymers with most APIs, including immunobiological substances, determines the relevance of using ISS as adjuvants for intranasal vaccination [3,23,24,25]. The administration of immunobiological drugs through the nasal cavity using adjuvant delivery systems allows both local immunization and systemic immune responses to be achieved. Due to the dense vascularization of the nasal mucosa and the uniform distribution of the dosage form, complete and prolonged absorption of IBD occurs [23,26,27,28].

Since 2020, due to the COVID-19 pandemic, speed of development has become the most important aspect of the IBD research and development (R&D) process. The rapid increase in SARS-CoV-2 infections required the fastest possible approach to developing and preparing a multi-million-dollar mass production of a new anti-coronavirus vaccine. The reality of the research laboratories and manufacturing sites revealed a lack of pharmaceutical development capacity for the IBD in a short timeframe. In the absence of a strategy for rapid development under the existing conditions, the process of new drug development becomes long-term and unproductive [29]. Quality, efficacy, and safety remain the primary goals of drug developers, given the importance of speed in pandemic drug development. To avoid further tragedies due to oversights in the drug lifecycle, a logical and consistent design of experiment (DoE) is required to accelerate development without sacrificing quality.

Challenges in ISS technology development and transfer can be addressed by applying the concept of “Quality by Design” (QbD) throughout the development of complex stimulus-responsive delivery systems. The QbD concept, unlike traditional single-factor analysis, incorporates quality risk management (QRM) and provides DoE planning that leads pharmaceutical development to optimal outcomes with significant reductions in financial, labor, and time costs [30].

Over the past three years, the QbD tool has been actively used by researchers to find and objectively justify vaccine formulations and technologies [31,32,33,34]. For example, in a study by van de Berg et al. [31], the design space for RNA vaccine synthesis was validated for the first time, which will facilitate the automation of rapid, high-quality RNA vaccine production. The work of Ghaemmaghamian, Z. et al. [33] also proposed and validated a QbD approach to justify the drying regime as one of the most important tools in vaccine stabilization.

The aim of the present work was to develop an adjuvant based on an ion-triggered in situ matrix for the intranasal delivery of virus-like particles, justified and carried out according to the QbD standard.

## 2. Materials and Methods

### 2.1. Instrumentation for Experiments

For the experiment, we used a magnetic stirrer (IKA C-mag Hs7 digital, Staufen im Breisgau, Germany), a pH meter (Starter 2100 pH Bench pH Meter ST2100-F, Shanghai, China), an autoclave (Tuttnauer, 3150 EL, Tel-Aviv, Israel), a coaxial cylinder viscometer (Lamy Rheology RM 200, Paris, France), and an in vitro model of the nasal cavity (developed at the Institute of Pharmacy named after A.P.Nelyubin, Sechenov University, Moscow, Russia).

### 2.2. Excipients

**Figure 1:**
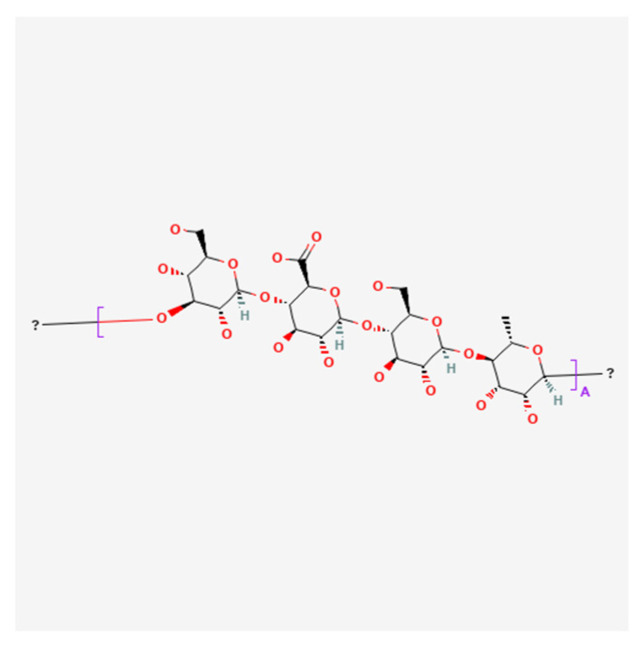
Gellan gum chemical structure (PubChem).

**Figure 2:**
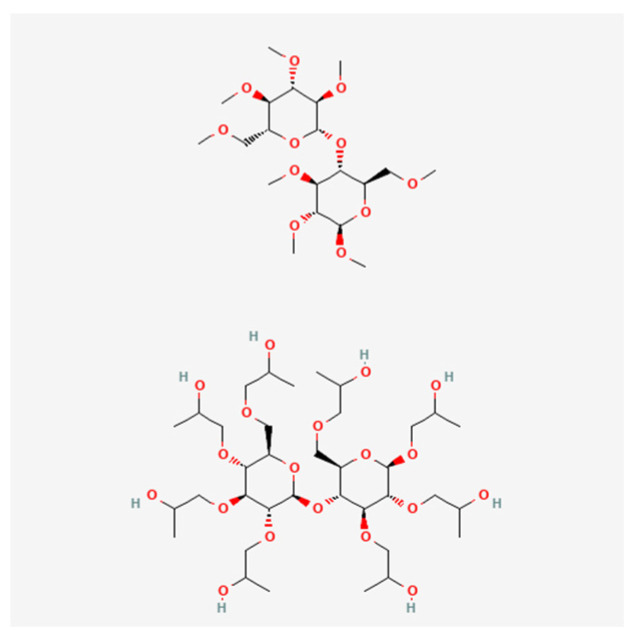
Hydroxypropyl methylcellulose chemical structure (PubChem).

**Figure 3:**
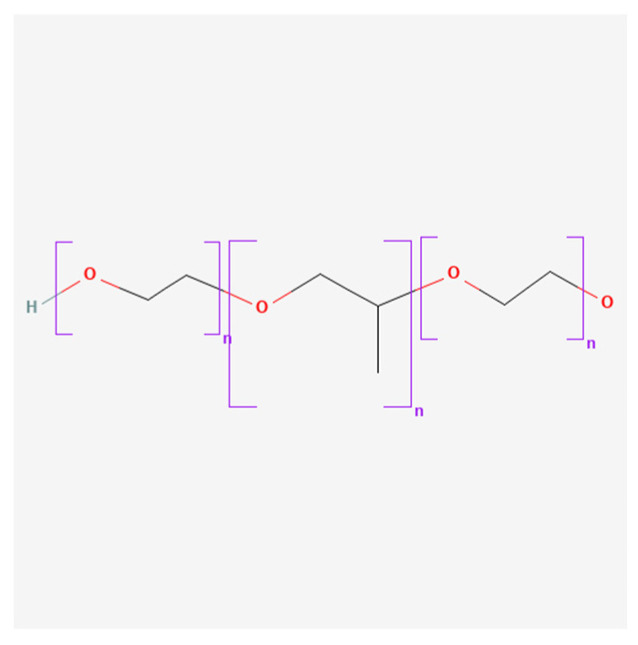
Poloxamer 124 chemical structure (PubChem).

The in situ ion-selective polymer used in this study was deacetylated gellan gum (GG) (Molecularmeal, Shanghai, China) (Figure 1), mucoadhesive polymers were hydroxypropyl methylcellulose (HPMC) M.W. = 86,000 Da (Ashland, Wilmington, DE, USA) (Figure 2), poloxamer 124 (Pol124)—44% poly(ethylene oxide), (PEO), M.W. = 2500 Da (Kolisolv^®^ P124 Geismar, BASF, Ludwigshafen am Rhein, Germany) (Figure 3), and water for injection as solvent. Phosphate buffer (PBS, pH = 6.8 ± 0.5) was added to some compositions to modify the in situ gel properties.

### 2.3. Model Immunobiologic Drug

Virus-like particles (VLP) obtained from the Gamaleya National Center for Epidemiology and Microbiology were used as the model IBP. VLPs were obtained according to “Recombinant virus-like particle for induction of specific immunity against severe acute respiratory syndrome virus SARS-CoV-2 and recombinant baculovirus for production of recombinant coronavirus proteins, Patent No. 2769224, priority from 20.12.2021”. The choice of doses was based on the previously obtained results of preclinical studies conducted in coordination with the Scientific Center for the Examination of Medical Products of the Ministry of Health of the Russian Federation.

### 2.4. Research Methodology

Samples of the tested matrices were obtained by dissolving polymers in purified water (compositions containing gellan gum were heated to a temperature of 80 °C). After the samples were stabilized for 24 h, they were sterilized by autoclaving at 124 °C for 30 min. Virus-like particles were introduced into the best compositions under aseptic conditions.

#### 2.4.1. Quality by Design (QbD) Methodology

**Figure 4:**
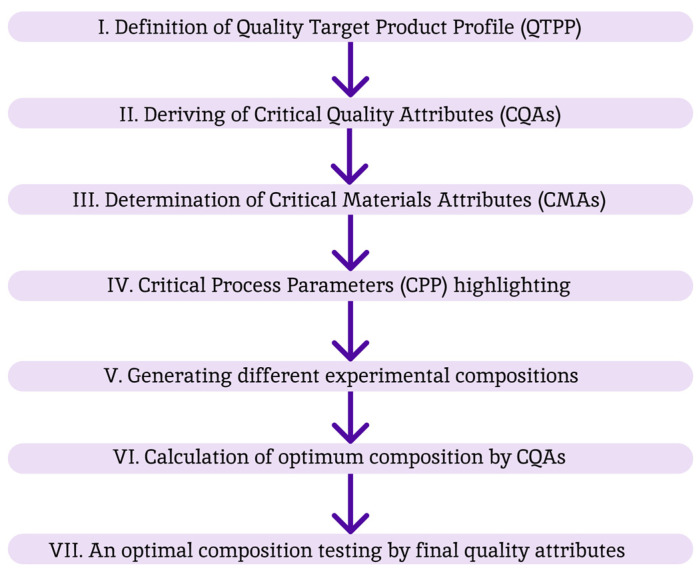
QbD methodology description.

The design of the experiment conducted using the QbD methodology is summarized in Figure 4.

As shown in Figure 1, the first step was to determine the Quality Target Product Profile (QTPP) of the developed ion-sensitive adjuvant for intranasal vaccine delivery (Table 1).

Then, based on the proposed and justified QTPP, critical quality attributes (CQA), critical material attributes (CMA) and critical process parameters (CPP) were identified. Their list is presented in Table 2.

To determine the influence of process parameters on critical quality attributes, formulations were prepared with variable process conditions: mixing time (“low”—10 min, “high”—60 min), mixing speed (“low”—50 rpm, “high”—500 rpm), structuring time (“low”—3 h, “high”—48 h), sterilization (yes/no). The next step was to determine the influence of the formulation components on the critical parameters. As critical parameters of the composition, the following were proposed: concentration of gelling agent—concentrations of 0.25% and 0.75% (m/V) were used as limit values; additional ingredient as which poloxamer 124, HPMC were used; concentration of additional ingredient (from 0.1% to 0.3% (m/V) for HPMC; from 1.0 to 4.0% (m/V) for poloxamer 124); solvent (water for injection, PBS in concentration from 5 to 15%); concentration of VLP—80 µg per dose.

The formulations obtained were tested for critical quality parameters, and the results were analyzed using MiniTab 17.0 software. The degree of influence of the factors was defined by the program as high (H), medium (M), or low (L).

#### 2.4.2. Determination of In Situ Gelling Ability

Depending on the stimulus for in situ gelation, each composition (1 mL) was placed under conditions similar to those found in the nasal cavity. Synthetic nasal secretion containing an equivalent physiological concentration of Na^+^, K^+^, and Ca^2+^ ions with a pH of 6.5 ± 0.5 was added to the ion-selective compositions.

#### 2.4.3. Measurement of pH

The polymer compositions were tested potentiometrically after preparation. Formulations with a pH critically low (<4.50) or critically high (>8.50) compared to the physiological pH of the nasal cavity (6.5 ± 0.5) were excluded from the study as they could cause ciliotoxic effects on the mucosa.

#### 2.4.4. Viscosity Measurements

Viscosity measurements were performed to determine the stability of the samples in the ash state after sterilization by autoclaving. The tests were performed at 20 °C, over a range of shear rates from 0 to 300 s^−1^ for 60 s. To obtain average results, three measurements were taken for each sample after half an hour of relaxation. The stability of the index was determined by calculating the plastic viscosity using the Casson model [39].

#### 2.4.5. Determination of the Completeness of Retention on the Mucosal Surface In Vitro

The study was carried out on an in vitro model of the nasal cavity. The model was irrigated with a mucin solution (~4%) containing nasal cavity ions (Na^+^, K^+^, Ca^2+^). The model was then wrapped in film to simulate the opposite side of the nasal cavity. 1 mL of the composition was injected into the cavity using a spray device (NEST Pre-filled Disposable Intranasal Atomization Device, Wuxi, China). To collect the sample exiting the model, a polymer collector was attached to the nasopharynx at the exit. The model was placed in a thermostat at 37 °C for 5 min (multiplicity of experiments = 5). In this way, the completeness of retention of the composition on the nasal mucosal surface was tested (1).

Completeness of retention = (1 mL − V_escaped from the model_) × 100% (1)

#### 2.4.6. Measurement of the Spray Torch

1 mL of the composition dyed with a water-soluble dye was sprayed onto a non-woven material on a flat horizontal surface at a distance of 5–7 cm. After spraying the non-woven material, the diameter of the circle was measured.

#### 2.4.7. In Vivo Tests

*Mesocricetus auratus* hamsters weighing approximately 100 g (Gamaleya National Centre for Epidemiology and Microbiology, Moscow, Russia) were selected for in vivo studies. The animals were selected and divided into 4 groups—2 control and 2 experimental—with 3 individuals in each group. All animals were housed according to the standards of the Ministry of Agriculture of the Russian Federation. They had unrestricted access to food and water and were treated in accordance with the Animal Welfare Act. The acute toxicity of the drug to the animal body was assessed on day 7 of the study.

## 3. Results and Discussions

A total of 14 placebo formulations of in situ gels based on ion-selective smart polymer (gellan gum) with added excipients were prepared. All compositions and polymer concentrations analyzed are listed in Table 3.

The generated compositions were tested according to the critical quality attributes selected in the QbD planning phase. When analyzing the influence of the process parameters (CPP) on the quality of the compositions, it was found that of all the parameters, only sterilization had a significant and meaningful effect on the CQA (Table 4). Therefore, all critical parameters of the composition set were further evaluated for stability after sterilization.

In the first stage of the screening, the stability of the stimulus sensitivity of the pool of formulations was assessed. The results are summarized in Table 5 and are marked with symbols, where “-” indicates no gel is formed; “+” indicates an unstructured gel is formed; “++” indicates a strong gel is formed and breaks down within 10 min; and “+++” indicates a strong gel is formed and retains its structure over time.

It was shown that compositions No. 2, 13, and 14, which initially had a high sensitivity to stimulants, completely retained it after sterilization by autoclaving. At the same time, it was observed that composition No. 3 acquired more pronounced properties of stimulus sensitivity after sterilization, and compositions No. 4 and 5 lost stimulus sensitivity insignificantly, remaining sufficiently selective to the artificial nasal fluid used in the experiment.

It is also worth noting that the formulations containing minimal concentrations of gellan gum and an additional ingredient—HPMC—as well as phosphate buffer as a co-solvent (No. 1, 8, 12) have the lowest stimulus sensitivity, which is also lost after sterilization.

This experiment demonstrated the feasibility of adding poloxamer 124 as an additional ingredient, as well as moderate amounts of phosphate buffer.

The next step was to determine the pH of each sample (Table 6). The pH ranged from an average of five measurements of 6.44 (sample No. 9) to 7.79 (sample No. 1). Thus, samples No. 1 and No. 9 did not meet the target profile, while the others were optimal in terms of pH and suitable for use as an adjuvant for intranasal VLP administration. Sterilization was shown to have a moderate effect on the pH, which remained within the normal range for the sample set.

The next phase of the study involved work on an in vitro model of the nasal cavity to determine the completeness of retention. Formulations No. 8 and 12 containing 0.25% gellan gum and 0.1% HPMC and 5% PBS, respectively, were shown to have the lowest retention capacity on the model. Meanwhile, the introduction of poloxamer 124 and phosphate buffer at a concentration higher than 5% was found to be effective in increasing the retention parameter. The concentration of ion-sensitive polymers also correlates with the retention parameters, which are confirmed by preliminary studies demonstrating the high mucoadhesive properties of gum.

It is also necessary to note the degree of influence of the sterilization stage on the retention parameter (Table 7). For some formulations, a significant increase in retention on the model was observed after sterilization by autoclaving (formulations No. 2, 3, and 14). At the same time, these formulations showed the highest increase in plastic viscosity after sterilization. Therefore, we can assume that in this case, the phenomenon of increased retention is related to the partial evaporation of the solvent during sterilization and the increase in the density of the polymer chains in the matrix.

For compound No. 7 (gellan gum content 0.25%, HPMC content 0.3%), a significant decrease in model retention capacity (by 20%) was observed. Further studies are required to explain this phenomenon.

It should also be noted that a large set of compositions were relatively stable (No. 1, 4, 5, 6, 8, 10, 11, 12, 13) for the parameters studied after sterilization, which makes these compositions promising for further study.

According to the parameters of the spray torch after sterilization (Table 8), only compositions No. 7, 9, 10, and 13 did not meet the target profile of the product quality—the index of the others met the optimal values.

Table 9 shows the results of the determination of the average (over three measurements) plastic viscosity using the Casson model for the set of experimental samples before and after sterilization (Table 9). The experimental data explain the changes in the properties and behavior of some compositions in the tests, the results of which were described earlier.

It should be noted that significant changes in plastic viscosity values after sterilization, according to the QTPP described above, were not strictly negative in the experiment—if they did not affect other critical quality attributes. The stability of plastic viscosity before and after sterilization, for example, characteristic of formulations No. 9, 10, and 12, does not guarantee the optimality of their other properties related to viscosity parameters (e.g., retention and spray pattern). Therefore, the viscosity values determined for the sample pool after sterilization—sufficient for optimal atomization through the syringe system—were taken into account. Compositions No. 2, 5, 6, and 14 became too viscous after sterilization for spraying and could not be considered further.

Already at the stage of studying the completeness of retention of placebo compositions, we can conclude that samples No. 7, 8, and 12 are unsuitable. Despite the good retention of composition No. 7 before sterilization, its index worsens after autoclaving (63%), which does not allow further use of the composition for in vivo tests. For compositions 8 and 12, the narrow nebulizer, which does not allow a complete distribution of the drug on the surface of the nasal mucosa, could be the main reason for the incomplete retention.

Based on the results of these tests, a comparison of the results of in vitro tests of various placebo compositions was performed, and the most suitable compositions No. 3, 4, and 9 with various additional ingredients were selected. The tested IBD was then added to these compositions. The summary results for all the parameters listed above are shown in Table 10, where the gelation ability is labeled as follows: “-” indicates no gel formed; “+” indicates an unstructured gel formed; “++” indicates a strong gel formed and broke down within 10 min; “+++” indicates a strong gel formed, maintaining its structure over time; and VLP titer stability: “+” indicates a stable titer, “-” indicates a significant decrease in titer.

From the summary data in Table 10, the advantage of compound No. 3 over the others is obvious in all the parameters analyzed. Therefore, it was proposed for further in vivo studies. Having demonstrated that there was no effect of IBD on the in situ gel, the studies were continued, and preliminary tests were performed on *Mesocricetus auratus* hamsters.

When the acute toxicity of the placebo and VLP formulations was evaluated, no external changes, behavioral changes, or changes in vital functions were observed.

The contribution of this study to the subsequent development of ion-sensitive adjuvants can be presented as an evaluation of the effect of composition on critical product quality parameters (Table 11).

**Figure 5:**
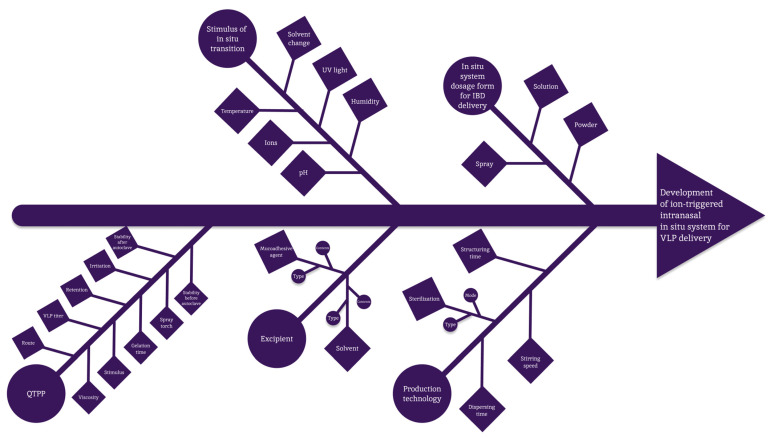
Ishikawa chart.

According to the results of the differential analysis, only the VLP concentration of 80 µg/dose did not significantly affect the critical quality parameters. Whereas both the concentration of the injected PBS, the type of additional ingredient (HPMC/poloxamer 124), and the concentration of gellan gum significantly changed the properties of the system (Figure 5).

It is curious that most researchers do not include such factors as the completeness of retention of the dosage form on the mucosal surface, the pH value, or the spray burner in the list of quality indicators. The rationale for the selection of these indicators in this study is simply to increase the speed of development by quickly screening unsuitable formulations using the basic, simplest indicators. If the investigational formulation is not able to adhere to the mucosa, has a non-physiological pH, and is not nebulized from the spray, then such a dosage form will be biopharmaceutically incorrect.

In a recent study by researchers at universities in India [10], a gellan gum-based delivery system for intranasal administration was developed to deliver an active ingredient (lorazepam) via a nose-to-brain mechanism. The scientists claim that a clear study design in line with the QbD concept led to excellent results. The correct study design allowed early elimination of inappropriate polymer formulations from the study and the selection of an excipient (carbopol 934) to enhance the performance of pure gellan gum.

Unlike many other studies, the article by Chen, Y. et al. examines the retention time of the drug on the surface of the nasal mucosa. This index is similar to the completeness of retention. In the study by Chinese scientists, the retention time was analyzed only in vivo without preliminary evaluation in in vitro models [11].

A recent study by Li, M. et al. demonstrates the potential use of MOFs as an antitumor drug delivery system through various methods of tumor exposure and depletion. The methods can be categorized into chemotherapy, photodynamic therapy, photothermal therapy, chemical kinetic therapy, sonodynamic therapy, immunotherapy, gene therapy, and starvation therapy [40]. Considering the novelty and relevance of this development, it cannot be said that such a solution is suitable as a delivery system for immunobiological substances for intranasal administration and not only. However, the modern trend of drug delivery by a nose-to-brain mechanism provides an opportunity to test the development of intranasal administration.

According to the results of the literature analysis, no analogues of the in situ matrix composition (GG + Pol124) proposed in this article have been developed for immunobiological drug delivery. In most of the existing developments in preclinical and clinical studies, the gel-forming polymers are thermoreversible polymers in combination with pH-sensitive and mucoadhesive ones.

## 4. Conclusions

Thus, the proposed QbD pharmaceutical development design allowed the identification of a composition containing 0.25% gellan gum and 4% poloxamer 124 as an optimal adjuvant composition with ion-selectivity towards artificial nasal fluid. After the introduction of VLP into the composition, it showed satisfactory results in terms of in vitro parameters (gelling ability, pH, retention completeness, spray torch).

## Figures and Tables

**Table 1:** Quality target product profile (QTPP).

Factor	Target	Justification
Route of administration	Intranasal	The intranasal route of administration among non-invasive delivery routes is one of the preferred routes of administration for SSRIs due to the stability and pharmacokinetics of these drugs. Nasal vaccines have numerous advantages over vaccines administered by invasive methods [26,35].
VLP administration	80 µg per dose	Preliminary immunogenicity studies of the developed preparation in various animal models showed that a pronounced immunogenic effect (activation of cellular and humoral immunity) was observed when the antigen was applied at a dose of 80 µg/dose.
pH	6.0–7.5	The optimal pH of the composition is due to the stability of the VLP [36].
Viscosity before gelation	<0.1 Pa∙s	The pre-phase transition viscosity is determined by the selected nebulizer system—NEST Pre-filled Disposable Intranasal Atomization Device (China)—to provide the required spray torch.
Gelation stimulus	Temperature 31–34 °C, nasal fluid composition 8.77 mg/mL NaCl, 2.98 mg/mL KCl, and 0.59 CaCl_2_ mg/mL (pH = 6 ± 0.1)	Gel conditions match the physiological norm of the nasal cavity and nasal secretions.
In situ gelation time	<60 s	This gelation time does not promote premature evacuation of the formulation from the nasal cavity into the nasopharynx—and subsequent loss of dose.
Viscosity stability after sterilization	Medium	The viscosity of polymer compositions based on non-starch polysaccharides may change after thermal sterilization. In this case, only the post-sterilization viscosity should be considered, which should comply with the viscosity requirements for in situ polymer solutions.
Post-sterilization stimulus sensitivity stability	High	It is essential for ion-sensitive compositions to maintain a high (sufficient) degree of sensitivity to the selected stimulus after sterilization.
Spray torch	>60 mm	In contrast to drugs intended for nose-to-brain delivery, a large mucosal irrigation area is important for intranasal vaccines, as this results in high bioavailability. However, it should be noted that too wide a spray pattern may contribute to dose loss.
Retention on the nasal cavity model	>80%	Similar retention percentages are characteristic of highly adhesive compositions, according to previously published studies on this model [37].
No irritating effect on mucosa	Required	Absence of ciliotoxic effects [38].
VLP titer stability	High	The components of the polymer matrix of the ion-sensitive adjuvant should be completely indifferent and should not affect the VLP titer or the efficacy of the administered vaccine.

**Table 2:** List of critical parameters.

CQA	CMA	CPP
Gelation stimulus	Gelling agent concentration	Sterilization
Solution viscosity	Additional ingredient	Stirring time
In situ gel viscosity	Type of solvent	Dispersion speed
Solution viscosity stability after sterilization	VLP concentration	Dispersing time
Spray torch		
pH		
Retention on in vitro model		

**Table 3:** Placebo compositions and polymer concentrations.

Sample No.	Gellan Gum Concentration, % (*m*/*v*)	Additional Excipient	Concentration of Additional Excipient, % (*m*/*v*)
**1**	0.25	-	-
**2**	0.75	-	-
**3**	0.25	Pol 124	4
**4**	0.25	Pol 124	1
**5**	0.75	Pol 124	4
**6**	0.75	Pol 124	1
**7**	0.25	HPMC	0.3
**8**	0.25	HPMC	0.1
**9**	0.75	HPMC	0.3
**10**	0.75	HPMC	0.1
**11**	0.25	PBS	15
**12**	0.25	PBS	5
**13**	0.75	PBS	15
**14**	0.75	PBS	5

**Table 4:** Results of determining the dependence of CQA on CPP.

	Gelation Stimulus	Solution Viscosity	Solution Viscosity Stability after Sterilization	Spray Torch	Retention on In Vitro Model
**Sterilization**	High	Low	High	High	High
**Stirring time**	Low	Low	Low	Low	Low
**Dispersion speed**	Low	Medium	Low	Low	Low
**Dispersing time**	Low	Low	Low	Low	Low

**Table 5:** Stability of stimulus responsiveness of in situ gelation of placebo formulations before and after sterilization (*n* = 5).

Sample No.	In Situ Gelation before Sterilization	In Situ Gelation after Sterilization
**1**	+	+
**2**	+++	+++
**3**	++	+++
**4**	+++	++
**5**	+++	++
**6**	++	+
**7**	++	+
**8**	+	-
**9**	++	++
**10**	++	++
**11**	++	+
**12**	+	-
**13**	+++	+++
**14**	+++	+++

**Table 6:** pH values of placebo formulations before and after sterilization (*n* = 5).

Sample No.	In Situ Gelation before Sterilization	In Situ Gelation after Sterilization
**1**	7.79	7.53
**2**	7.34	7.16
**3**	7.47	7.61
**4**	7.52	7.63
**5**	7.27	7.32
**6**	7.32	7.02
**7**	7.49	7.11
**8**	7.62	6.96
**9**	6.44	6.84
**10**	6.58	6.89
**11**	6.70	7.00
**12**	6.69	7.35
**13**	6.75	7.00
**14**	6.80	7.20

**Table 7:** Completeness of retention of placebo formulations on the surface of an in vitro model of the nasal cavity before and after sterilization (*n* = 5).

Sample No.	Retention Completeness before Sterilization, %	Retention Completeness after Sterilization, %
**1**	73	77
**2**	78	93
**3**	80	93
**4**	73	78
**5**	87	82
**6**	85	85
**7**	83	63
**8**	53	50
**9**	90	80
**10**	87	85
**11**	77	81
**12**	50	53
**13**	90	98
**14**	77	98

**Table 8:** Spray torch of placebo formulations before and after sterilization (*n* = 5).

Sample No.	Spray Torch before Sterilization, mm	Spray Torch after Sterilization, mm
**1**	105	75
**2**	75	65
**3**	65	60
**4**	65	65
**5**	70	60
**6**	60	75
**7**	50	55
**8**	50	60
**9**	40	45
**10**	50	50
**11**	50	60
**12**	50	70
**13**	70	50
**14**	50	65

**Table 9:** Plastic viscosity of placebo formulations before and after sterilization (*n* = 3).

Sample No.	Average Plastic Viscosity According to Casson Model before Sterilization, Pa*s	Average Plastic Viscosity According to Casson Model after Sterilization, Pa*s
**1**	0.0365	0.0645
**2**	0.0457	0.1342
**3**	0.0079	0.0541
**4**	0.0274	0.0456
**5**	0.0275	0.1389
**6**	0.0127	0.1347
**7**	0.0275	0.0413
**8**	0.0234	0.0397
**9**	0.0597	0.0598
**10**	0.0328	0.0356
**11**	0.0197	0.0297
**12**	0.0222	0.0278
**13**	0.0446	0.0732
**14**	0.0278	0.1179

**Table 10:** Results of in vitro studies of the optimal set of formulations containing VLPs.

Attribute Name	Sample No. 3	Sample No. 4	Sample No. 9
**In situ gelling ability**	+++	++	++
**pH**	7.34	7.63	6.84
**Retention completeness, %**	90	78	80
**Spray torch, mm**	70	65	45
**VLP titer stability**	+	+	+

**Table 11:** Results of determining the dependence of CQA on CPP.

	Gelation Stimulus	Solution Viscosity	Solution Viscosity Stability after Sterilization	Spray Torch	Retention on In Vitro Model
**Sterilization**	High	High	High	High	Medium
**Stirring time**	High	High	High	High	Medium
**Dispersion speed**	High	High	High	Medium	High
**Dispersing time**	Low	Low	Low	Low	Low

## Data Availability

The data presented in this study are openly available in the article.

## References

[1] Nagaraja S., Basavarajappa G.M., Karnati R.K., Bakir E.M., Pund S. (2021). Ion-Triggered in Situ Gelling Nanoemulgel as a Platform for Nose-to-Brain Delivery of Small Lipophilic Molecules. Pharmaceutics.

[2] Zhang Y., Li Q., Hu J., Wang C., Wan D., Li Q., Jiang Q., Du L., Jin Y. (2022). Nasal Delivery of Cinnarizine Thermo-and Ion-Sensitive In Situ Hydrogels for Treatment of Microwave-Induced Brain Injury. Gels.

[3] Teaima M.H., Helal D.A., Alsofany J.M., El-Nabarawi M.A., Yasser M. (2022). Ion-Triggered In Situ Gelling Intranasal Spray of Dronedarone Hydrochloride Nanocarriers: In Vitro Optimization and In Vivo Pharmacokinetic Appraisal. Pharmaceutics.

[4] Demina N.B., Bakhrushina E.O., Bardakov A.I., Krasnyuk I.I. (2019). Design of Intranasal Dosage Forms: Biopharmaceutical Aspects. Farmaciya (Pharmacy).

[5] Safonova E.A., Razina T.G., Zueva E.P., Lopatina K.A., Efimova L.A., Gur’ev A.M., Rybalkina O.Y., Khotimchanko Y.S. (2012). Prospects for the Use of Plant Polysaccharides in Complex Treatment of Malignant Tumors. Eksperimental’naya i Klinicheskaya Farmakologiya.

[6] Raquel Maia F., Correlo V.M., Oliveira J.M., Reis R.L. (2019). Natural Origin Materials for Bone Tissue Engineering: Properties, Processing, and Performance. Principles of Regenerative Medicine.

[7] Khotimchenko M., Khozhaenko E., Kolenchenko E., Khotimchenko Y. (2012). Influence of Pectin Substances on Strontium Removal in Rats. Int. J. Pharm. Pharm. Sci..

[8] Marcotuli I., Colasuonno P., Hsieh Y.S.Y., Fincher G.B., Gadaleta A. (2020). Non-Starch Polysaccharides in Durum Wheat: A Review. Int. J. Mol. Sci..

[9] Sztupecki W., Rhazi L., Depeint F., Aussenac T. (2023). Functional and Nutritional Characteristics of Natural or Modified Wheat Bran Non-Starch Polysaccharides: A Literature Review. Foods.

[10] Shah V., Sharma M., Pandya R., Parikh R.K., Bharatiya B., Shukla A., Tsai H.-C. (2017). Quality by Design Approach for an in Situ Gelling Microemulsion of Lorazepam via Intranasal Route. Mater. Sci. Eng. C.

[11] Chen Y., Liu Y., Xie J., Zheng Q., Yue P., Chen L., Hu P., Yang M. (2020). Nose-to-Brain Delivery by Nanosuspensions-Based in Situ Gel for Breviscapine. Int. J. Nanomed..

[12] Fahmy U., Badr-Eldin S., Ahmed O., Aldawsari H., Tima S., Asfour H., Al-Rabia M., Negm A., Sultan M., Madkhali O. (2020). RETRACTED: Intranasal Niosomal In Situ Gel as a Promising Approach for Enhancing Flibanserin Bioavailability and Brain Delivery: In Vitro Optimization and Ex Vivo/In Vivo Evaluation. Pharmaceutics.

[13] Cai Z., Song X., Sun F., Yang Z., Hou S., Liu Z. (2011). Formulation and Evaluation of In Situ Gelling Systems for Intranasal Administration of Gastrodin. AAPS PharmSciTech.

[14] Stoppel W.L., White J.C., Horava S.D., Henry A.C., Roberts S.C., Bhatia S.R. (2014). Terminal Sterilization of Alginate Hydrogels: Efficacy and Impact on Mechanical Properties. J. Biomed. Mater. Res. B Appl. Biomater..

[15] Carranza T., Zalba-Balda M., Baraibar M.J.B., de la Caba K., Guerrero P. (2022). Effect of Sterilization Processes on Alginate/ Gelatin Inks for Three-Dimensional Printing. Int. J. Bioprint..

[16] Sanap S.N., Bisen A.C., Kedar A., Yadav K.S., Krishna A., Akhir A., Chopra S., Mugale M.N., Bhatta R.S. (2022). Chitosan/HPMC-Based Mucoadhesive Film Co-Loaded with Fluconazole and Ofloxacin for Management of Polymicrobial Keratitis. Int. J. Biol. Macromol..

[17] Islam M., Park T.-E., Reesor E., Cherukula K., Hasan A., Firdous J., Singh B., Kang S.-K., Choi Y.-J., Park I.-K. (2015). Mucoadhesive Chitosan Derivatives as Novel Drug Carriers. Curr. Pharm. Des..

[18] Bakhrushina E.O., Mikhel I.B., Pyzhov V.S., Demina N.B., Krasnyuk I.I., Krasnyuk I.I. (2023). Development of In Situ Intranasal System Based on Chitosan Formate. Bull. Exp. Biol. Med..

[19] Cho C.S., Hwang S.K., Gu M.J., Kim C.G., Kim S.K., Ju D., Yun C.H., Kim H.J. (2021). Mucosal Vaccine Delivery Using Mucoadhesive Polymer Particulate Systems. Tissue Eng. Regen. Med..

[20] Fahmy R.H. (2012). Statistical Approach for Assessing the Influence of Calcium Silicate and Hpmc on the Formulation of Novel Alfuzosin Hydrochloride Mucoadhesive-Floating Beads as Gastroretentive Drug Delivery Systems. AAPS PharmSciTech.

[21] Yong C.S., Oh Y.-K., Kim Y.-I., Kim J.O., Yoo B.-K., Rhee J.-D., Lee K.C., Kim D.-D., Park Y.-J., Kim C.-K. (2005). Physicochemical Characterization and in Vivo Evaluation of Poloxamer-Based Solid Suppository Containing Diclofenac Sodium in Rats. Int. J. Pharm..

[22] Choi H.G., Oh Y.K., Kim C.K. (1998). In Situ Gelling and Mucoadhesive Liquid Suppository Containing Acetaminophen: Enhanced Bioavailability. Int. J. Pharm..

[23] Bakhrushina E.O., Mikhel I.B., Kondratieva V.M., Demina N.B., Grebennikova T.V. (2022). In Situ Gels as a Modern Method of Intranasal Vaccine Delivery. Probl. Virol..

[24] Özkan B., Altuntaş E., Ünlü Ü., Doğan H.H., Özsoy Y., Çakır Koç R. (2023). Development of an Antiviral Ion-Activated In Situ Gel Containing 18β-Glycyrrhetinic Acid: A Promising Alternative against Respiratory Syncytial Virus. Pharmaceutics.

[25] Taymouri S., Shahnamnia S., Mesripour A., Varshosaz J. (2021). In Vitro and in Vivo Evaluation of an Ionic Sensitive in Situ Gel Containing Nanotransfersomes for Aripiprazole Nasal Delivery. Pharm. Dev. Technol..

[26] Bakhrushina E.O., Mikhel I.B., Kondratieva V.M., Demina N.B., Grebennikova T.V., Krasnyuk I.I., Krasnyuk I.I. (2023). Main Aspects of Pharmaceutical Development of In Situ Immunobiological Drugs for Intranasal Administration. Curr. Pharm. Biotechnol..

[27] Ball J.P., Springer M.J., Ni Y., Finger-Baker I., Martinez J., Hahn J., Suber J.F., DiMarco A.V., Talton J.D., Cobb R.R. (2017). Intranasal Delivery of a Bivalent Norovirus Vaccine Formulated in an in Situ Gelling Dry Powder. PLoS ONE.

[28] Park J.S., Oh Y.K., Yoon H., Kim J.M., Kim C.K. (2002). In Situ Gelling and Mucoadhesive Polymer Vehicles for Controlled Intranasal Delivery of Plasmid DNA. J. Biomed. Mater. Res..

[29] Kis Z., Kontoravdi C., Dey A.K., Shattock R., Shah N. (2020). Rapid Development and Deployment of High-volume Vaccines for Pandemic Response. J. Adv. Manuf. Process..

[30] Smekhova I.E., Shigarova L.V., Andreeva P.I., Flisyuk E.V., Dzyuba A.S. (2022). Application of Quality-by-Design Approach to Justify the Composition and Technology of Two-Component Suppositories. Drug Dev. Regist..

[31] van de Berg D., Kis Z., Behmer C.F., Samnuan K., Blakney A.K., Kontoravdi C., Shattock R., Shah N. (2021). Quality by Design Modelling to Support Rapid RNA Vaccine Production against Emerging Infectious Diseases. npj Vaccines.

[32] Daniel S., Kis Z., Kontoravdi C., Shah N. (2022). Quality by Design for Enabling RNA Platform Production Processes. Trends Biotechnol..

[33] Ghaemmaghamian Z., Zarghami R., Walker G., O’Reilly E., Ziaee A. (2022). Stabilizing Vaccines via Drying: Quality by Design Considerations. Adv. Drug Deliv. Rev..

[34] Hocharoen L., Noppiboon S., Kitsubun P. (2021). Toward QbD Process Understanding on DNA Vaccine Purification Using Design of Experiment. Front. Bioeng. Biotechnol..

[35] Nagai M., Moriyama M., Ichinohe T. (2021). Oral Bacteria Combined with an Intranasal Vaccine Protect from Influenza a Virus and Sars-Cov-2 Infection. mBio.

[36] Zoratto S., Heuser T., Friedbacher G., Pletzenauer R., Graninger M., Marchetti-Deschmann M., Weiss V.U. (2023). Adeno-Associated Virus-like Particles’ Response to PH Changes as Revealed by NES-DMA. Viruses.

[37] Bakhrushina E.O., Shulikina D.S., Mikhel I.B., Demina N.B., Krasnyuk I.I. (2023). Development and Aprobation of In Vitro Model of Nasal Cavity for Stanadartisation of In Situ Drug Delivery Systems. Proc. Voronezh State Univ..

[38] Li C., Li C., Liu Z., Li Q., Yan X., Liu Y., Lu W. (2014). Enhancement in Bioavailability of Ketorolac Tromethamine via Intranasal in Situ Hydrogel Based on Poloxamer 407 and Carrageenan. Int. J. Pharm..

[39] Bakhrushina E.O., Khodenok A.I., Pyzhov V.S., Solomatina P.G., Demina N.B., Korochkina T.V., Krasnyuk I.I. (2023). Study of the Effect of Active Pharmaceutical Ingredients of Various Classes of BCS on the Parameters of Thermosensitive Systems Based on Poloxamers. Saudi Pharm. J..

[40] Li M., Zhang Z., Yu Y., Yuan H., Nezamzadeh-Ejhieh A., Liu J., Pan Y., Lan Q. (2023). Recent Advances in Zn-MOFs and Their Derivatives for Cancer Therapeutic Applications. Mater. Adv..

